# Psychological impact of the COVID-19 pandemic on dental health personnel in Norway

**DOI:** 10.1186/s12913-021-06443-y

**Published:** 2021-05-03

**Authors:** M. M. Uhlen, V. E. Ansteinsson, L. Stangvaltaite-Mouhat, L. Korzeniewska, R. Skudutyte-Rysstad, M. Shabestari, I. Mdala, E. A. S. Hovden

**Affiliations:** 1 Oral Health Centre of Expertise in Eastern Norway (OHCE-E), Oslo, Norway; 2 Department of Clinical Dentistry, Faculty of Health Sciences, UiT - The Arctic University of Norway, Tromsø, Norway; 3 General Practice Research Unit, Department of General Practice, Institute of Health and Society, University of Oslo, Oslo, Norway

## Abstract

**Background:**

The SARS-CoV-2 pandemic put a pressure on all healthcare professionals and has affected the delivery of health care services globally. There is a need to understand the impact on different health care professionals in different countries. The aim of the present study was to explore the psychological impact of the pandemic among dental staff in Norway in relation to background characteristics, work situation and preparedness of the service.

**Methods:**

A structured questionnaire sent electronically to dentists, dental hygienists and dental assistants inquired information about the lockdown period in Norway (13 March-17 April 2020). Distributions of background characteristics, perceptions of preparedness and psychological impact were calculated. Exploratory factor analysis was performed, and Structural Equation Models (SEMs) were used to compare psychological impact between dental professionals treating patients versus not during lockdown.

**Results:**

Among the 1237 respondents, 58.8% worked clinically with patients. The majority were concerned of becoming infected (71.9%), of infecting others (85.4%) and/or of their family becoming infected (76.9%). Respondents who treated patients felt significantly more insecure about whether having become infected or not. The minority felt discriminated (6.7%), worried about death (11.7%), felt that life was threatening (9.8%) or felt loss of control of their lives (8.9%). More than 80% agreed that their workplace handled the situation well. Four factors were retrieved from the factor analysis. SEMs showed that gender and work experience had a significant effect on the factors Instability, Infection and Concerns. Respondents with work experience ≥10 years were less likely to express fear about Instability and Infection. Personnel reporting that their workplace had adequate equipment were also less concerned, however having adequate equipment did not reduce the factor Loss of control.

**Conclusion:**

The present study showed a considerable psychological impact of the COVID-19 pandemic on dental personnel in Norway regardless of working clinically with patients or not. However, working with patients increased the insecurity about own infection status and of infecting people close to them. A safe working environment and adequate infection control measures are associated with less fear of infection and feeling of instability.

## Introduction

The coronavirus disease 2019 pandemic (COVID-19), caused by severe acute respiratory syndrome coronavirus 2 (SARS-CoV-2), is an unprecedented situation that has affected the population globally and generated an emergency status in health systems worldwide [1] including the dental health service system [2, 3].

On 12 March 2020, a national lockdown was announced by the Norwegian government, and during the lockdown (13 March – 17 April), dental health services postponed routine non-urgent dental health care. The Norwegian Directorate of Health requested the dental public sector to establish an emergency dental service for patients with suspected or confirmed COVID-19, and specific public clinics were designated to provide urgent treatment for these patients. Norway has not had a similar virus outbreak in the past and did not have national recommendations for infection prevention and control in dental practice before 2018 [2]. According to a recent questionnaire study among dental health personnel, oral health care has been managed relatively well in this period. However, the sudden increase in demand of personal protective equipment (PPE) caused a shortage of PPE in the dental service, and several deviations from procedures were reported. In addition, less than half of the respondents felt that their workplace was well equipped to handle an escalation of the situation [2].

The practice of dentistry involves close contact with patients and the use of rotary and surgical instruments that create a visible spray containing droplets of water, saliva, blood, microorganisms and other debris [4, 5]. SARS-CoV-2 transmission during dental procedures may happen through the inhalation of aerosol/droplets from infected individuals or direct contact with mucous membranes, oral fluids, and contaminated instruments and surfaces. Dental health personnel are thus at high risk of contagion when performing routine dental procedures, and the dental office may serve as a cross-infection location if adequate precautions are not taken. In addition, as individuals with COVID-19 may be asymptomatic for several days, they pose a risk to the dental health personnel when seeking dental treatment [6]. The role of dental professionals in preventing transmission of SARS-CoV-2 is therefore critically important [7–9].

Data from previous epidemics show that highly infectious diseases may have a significant impact on mental health, and increased anxiety and stress-related symptoms of both patients and health workers have been reported [10]. Due to the COVID-19 pandemic, health personnel worldwide are encountering constant stress during their daily work, related to risk of infection, frustration, exhaustion and social isolation [11]. It is therefore reasonable to assume that the health consequences of the pandemic are not limited to those directly related to own infection.

Investigation of the impacts of COVID-19 on mental health has been identified as a high research priority [12]. Understanding the psychological impact in different populations can provide a theoretical basis for the identification of individuals at risk and for designing interventions. This information may also contribute to the development of government policies and dedication of resources, which are of critical importance for public health at a global level [13]. Perceived vulnerability has been shown to be positively related to COVID-19-related worries, social isolation and traumatic stress [10]. Due to the possible high risk of contagion when performing routine dental procedures and the close relationship between risk of infection and psychological stress and anxiety, it is essential that this aspect is recognized also in the dental health services. Knowledge on the psychological impact of the pandemic on dental personnel is important both to facilitate for an optimal treatment of patients as well as for the psychological wellbeing of professionals. However, studies investigating COVID-19 outbreak related concerns and emotional reactions among dental staff from different countries and populations are still required. It is not known how the dental personnel in Norway experienced the situation on a subjective and psychological level. Therefore, the aim of the present study was to explore the specifics on factors that influence the psychological impact among dental personnel in relation to background characteristics, their perceived work situation and preparedness of the service in the lockdown period due to COVID-19 in Norway.

## Materials and methods

### Study design and participants

The participants of the present study were dental professionals (dental specialists, general dental practitioners, dental hygienists and dental assistants) recruited in May 2020, after the lockdown period caused by the COVID-19 pandemic in Norway.

A structured questionnaire was sent electronically via QuestBack to chief dental officers in all counties in Norway, who were asked to distribute the questionnaire among public dental clinics. Invitations to dentists in the private sector were distributed via local units of the Norwegian Dental Association (NDA). Three reminders for participation were sent, and the data collection ended on 26 June 2020.

### Questionnaire

The self-reported questionnaire was constructed based on information provided by Centers for Disease Control and Prevention (CDC), World Health Organization (WHO), Norwegian Institute of Public Health, Ministry of Health in Norway, guidelines provided by the Norwegian counties, and on previous research conducted under the SARS epidemic in 2002–2003 [2, 14]. The questionnaire consisted of 4 parts: I) Background characteristics, II) Dental health service management, including treatment of patients suspected or confirmed to have COVID-19, III) Dental staff perception of risk and preparedness, and IV) Psychological impact. The management of urgent dental care and perception of risk and preparedness of the service were discussed in a previously published study [2], while the current study was predefined to report the results from the first, third and fourth part of the questionnaire to explore the psychological impact among dental personnel. The background characteristics included in the present study are gender, work experience in years (dichotomized into ≤9 years and ≥ 10 years), profession (dentist, dental hygienist, dental assistant), size of dental clinic (large ≥7 employees, and small <7 employees), sector of main workplace (public, private), and if the respondent worked clinically with patients during COVID-19 outbreak (yes/no) (Table 1). Dental staff perception of risk and preparedness included four statements concerning respondents’ workplace preparedness to treat patients suspected or confirmed to have COVID-19, preparedness in COVID-19 lockdown period, preparedness to possible escalation of COVID-19 pandemic and respondents’ perceptions on risk of infection. Responses were scored on a 5-point Likert scale (from 1 completely agree to 5 completely disagree) and dichotomized into agree/completely agree versus undecided/disagree/completely disagree) (Table 2). The incidence of cases in counties was retrieved from Norwegian Institute of Public Health, and subsequently the counties were grouped into low incidence counties (< 100 reported cases per 100,000), medium incidence counties (100–150 reported cases per 100,000) and high incidence counties (> 150 reported cases per 100,000) for statistical analyses [15].

**Table 1:** Background characteristics of respondents

Characteristics	Worked clinically with patients		
	Yes *n* = 727 (58.8%)	No *n* = 510 (41.2%)	Total *n* = 1237 (100%)
Gender*			
Female	636 (87.5%)	470 (92.2%)	1106 (89.4%)
Male	91 (12.5%)	40 (7.8%)	131 (10.6%)
**Work experience (years)**			
0–9	240 (33.0%)	149 (29.2%)	389 (31.4%)
≥ 10	487 (67.0%)	361 (70.8%)	848 (68.6%)
Profession*			
Dentist	413 (56.8%)	177 (34.7%)	590 (47.7%)
Dental hygienist	66 (9.1%)	169 (33.1%)	235 (19%)
Dental assistant	248 (34.1%)	164 (32.2%)	412 (33.3%)
**Size of dental clinic**			
Small (< 7 employees)	164 (22.6%)	129 (25.3%)	293 (23.7%)
Large (≥ 7 employees)	563 (77.4%)	381 (74.7%)	944 (76.3%)
**Work sector**			
Public	664 (91.3%)	470 (92.2%)	1134 (92%)
Private	63 (8.7%)	40 (7.8%)	103 (8%)

*p < 0.05 differences between dental professionals working and not working clinically with patients in the lockdown period

**Table 2:** Respondents’ perceptions of workplace preparedness for the COVID-19 pandemic

Characteristics	Worked clinically with patients		
	Yes *n* = 727 (58.8%)	No *n* = 510 (41.2%)	Total *n* = 1237 (100%)
Is your clinic designated to treat patients suspected or confirmed to have COVID-19?*			
Yes	114 (15.7%)	56 (11.0%)	170 (13.7%)
No	613 (84.3%)	454 (89.0%)	1067 (86.3%)
**My workplace has currently adequate infection control equipment**			
Agree/completely agree	456 (62.7%)	310 (60.8%)	766 (61.9%)
Undecided/disagree/completely disagree	271 (37.3%)	200 (39.2%)	471 (38.1%)
**My workplace handles the current situation well**			
Agree/completely agree	597 (82.1%)	438 (85.9%)	1035 (83.7%)
Undecided/disagree/completely disagree	130 (17.9%)	72 (14.1%)	202 (16.3%)
**My workplace is well equipped to handle an escalation of COVID-19 situation**			
Agree/completely agree	289 (39.8%)	212 (41.6%)	501 (40.5%)
Undecided/disagree/completely disagree	438 (60.2%)	298 (58.4%)	736 (59.5%)

*p < 0.05 differences between dental professionals working and not working clinically with patients in the lock-down period

Part IV was based on the fear scale developed by Ho et al. [14] under SARS epidemic, originally developed to investigate the nature of fear among health care workers during the SARS pandemic in 2002–2003, and to establish an inventory for measuring such fear for future outbreaks. The fear scale was adapted to the COVID-19 outbreak in Norway and its following lockdown between 13 March – 17 April 2020. The text was translated into Norwegian and back translated into English. It consisted of 18 items including fear of becoming infected, fear of infecting others, fear of family members becoming infected, fear related to working with Covid-19 patients, and fear of death (Table 3). Dental staff was asked to respond to each of the 18 items on a 4-point Likert scale (0 definitely false, 1 somewhat false, 2 somewhat true, 3 definitely true) to assess the 18-items. For statistical analyses the responses were dichotomized into “disagree” (points 0–1) and “agree” (points 2–3).

**Table 3:** Frequency distributions of dental personnel (dentists, dental hygienists and dental assistants) responding “agree” and “strongly agree” to the COVID-19 questionnaire items

COVID-19 Item	Worked clinically with patients	
	Yes ***n*** = 727	No ***n*** = 510
COVID-19 makes me:	**%** **agree/strongly agree**	**%** **agree/strongly agree**
Fear that I will be infected	72,2	71,4
Fear that I will infect others	87,2	82,9
Feel insecure about whether I have been infected or not*	56,9	49,4
Feel that the virus is very close to me and that it can invade my body at any time	24,2	22,9
Feel very insecure	30,7	37,3
Feel that life is threatening	9,8	9,4
Feel that I have lost control of my life	8,9	8,0
Think of death / to die	11,7	10,0
Feel that the virus will get out of control and spread continuously	33,1	32,7
Worry about whether my family will be infected	77,7	75,7
Dream that my family or my colleagues are infected*	18,8	12,5
Fear that I will end up in quarantine or be forced to limit my activities	43,3	39,0
Worry about increased work pressure	43,9	44,7
Feeling discriminated against by others	6,7	6,3
Worry about whether my family or friends will keep me at a distance because of my job responsibilities	21,3	23,3
Worry about having to work with Covid-19 patients	44,3	45,7
Worry about other health problems in myself	27,6	32,9
Worry about other health problems in my family members*	61,1	59,0

*p < 0.05 differences between dental professionals working and not working clinically with patients in the lock-down period

The questionnaire was face validated by several dental health service managers and pre-tested by 10 dentists, whose responses were not included in the analysis.

### Statistical analysis

Frequency distributions were used for descriptive statistics. Chi-square test was applied to compare differences in the frequency distribution between dental professionals who worked clinically with patients and those who did not in the lockdown period in Norway. The level of statistical significance was set at 5%.

We carried out an explanatory factor analysis (EFA) on the 18 items using the oblique rotation (in particular, the direct oblimin) to identify latent constructs. The adequacy of the data for factor analysis (FA) was determined from measures of sampling adequacy: Kaiser-Meyer-Olkin (KMO) and Battlett’s test of sphericity. We used a Cronbach’s alpha test to determine the internal consistency of the 18 items of the COVID-19 questionnaire. A Cronbach’s alpha estimate ≥0.70 was considered acceptable (Kline, 1999). The Kaiser criterion of retaining factors with eigenvalues greater than 1 was applied. Factor scores of the identified latent variables were summed to form a total score of COVID-19 concerns. Further, we used structural equation models (SEMs) to model and understand the relationship between working with patients when restrictions were tight and the latent constructs. The SEMs were adjusted for socio-demographic factors and work-related factors. Descriptive analyses and factor analysis were carried out using IBM SPSS Statistics 26 whereas StataSE 16 was used to conduct SEMs.

### Ethical considerations

Approval was obtained from the Norwegian Centre for Research Data (907304). Voluntary participation was based on an electronically signed informed consent.

## Results

### Participants/respondents

Seven hundred and twenty-seven (58.8%) of the 1237 dental professionals who participated in this study worked clinically with patients during the period during the lockdown period (Table 1). This represents approximately 10% of dental professionals in Norway and distribution of responders in relation to professional category was representative to distribution in the country [16]. A significantly higher number of female dental professionals participated in the study (1106 (89.4%)) compared to males (131 (10.6%)). The results showed a greater percentage of dentists engaged in patient care during the pandemic, compared to dental hygienists. More than 60% of the respondents had more than 10 years of clinical experience. Furthermore, the majority of the respondents worked at large clinics with more than 7 employees, in the public dental service sector (Table 1).

### Perceptions of preparedness for the COVID-19 pandemic

Of the 727 respondents that worked clinically with patients in the lockdown period, only 15.7% worked at clinics designated to treat patients suspected or confirmed to have COVID-19. The majority of the respondents agreed or completely agreed that dentists, dental hygienists and dental assistants are at high risk of contracting COVID-19. This was regardless of whether respondents worked clinically with patients or not in the lockdown period. More than half of the respondents agreed that their workplace had adequate equipment to prevent a possible infection with COVID-19, and more than 80% of the respondents agreed that their workplace handled the situation during the lockdown period well. However, less than half agreed that their workplace was well equipped to handle an escalation of COVID-19 situation (Table 2).

### Psychological impact of the COVID-19 pandemic

The majority of the respondents were concerned that they would be infected (*n* = 889, 71.9%), that they would infect others (*n* = 1057, 85.4%) or that their family would be infected (*n* = 951, 76.9%). This was regardless of whether the respondents worked clinically with patients or not in the lockdown period (Table 3).

Respondents working clinically with patients in the lockdown period felt significantly more insecure about whether they had been infected or not (*n* = 414, 56.9%). Furthermore, the same respondents had significantly higher general concerns about other health problems in their family members as a consequence of the pandemic (*n* = 444, 61.1%) and reported dreaming that their family or colleagues were infected (*n* = 137, 18.8%) (Table 3).

The minority of the respondents felt discriminated by others (*n* = 49, 6.7%), were worrying about death (*n* = 85, 11.7%), felt loss of control of their lives (*n* = 65, 8.9%) and that life was threatening (*n* = 71, 9.8%) (Table 3).

### Extraction of factors using the explanatory factor analysis)

The Kaiser-Meyer-Olkin (KMO) statistic suggested that the sample size was adequate for Factor Analysis (KMO = 0.92). We extracted four factors with eigenvalues greater that the Kaiser criterion of 1 and these factors explained 57.4% of the variance. Factor loadings less than 0.40 were excluded from further analysis (Table 4). A scree plot (not shown) also suggested retaining four factors. The four factors extracted from the items were labeled; 1. *Instability,* 2; *Infection 3; Loss of control* and *4; Concerns.*

**Table 4:** Summary results of an exploratory factor analysis of the COVID-19 questionnaire. Factor loadings less than 0.40 are not presented (*N* = 1237)

		Factor loadings rotated			
	COVID-19 Item	Factor 1	Factor 2	Factor 3	Factor 4
	COVID-19 makes me:				
**Factor 1** **Instability**	Dream that my family or my colleagues are infected	0.43			
	Fear that I will end up in quarantine or be forced to limit my activities	0.44			
	Worry about increased work pressure	0.46			
	Feeling discriminated against by others	0.48			
	Worry about whether my family or friends will keep me at a distance because of my job responsibilities	0.65			
**Factor 2** **Infection**	Fear that I will be infected		0.49		
	Fear that I will infect others		0.50		
	Worry about whether my family will be infected		0.63		
	Worry about having to work with Covid-19 patients		0.43		
	Worry about other health problems in my family members		0.65		
**Factor 3** **Loss of control**	Feel that life is threatening			−0.88	
	Feel that I have lost control of my life			−0.74	
	Think of death / to die			−0.67	
**Factor 4** **Concerns**	Feel insecure about whether I have been infected or not				0.63
	Feel that the virus is very close to me and that it can invade my body at any time				0.45
	Eigenvalues	6.64	1.49	1.20	1.003
	Percent (%) of variance	36.89	8.25	6.65	5.57
	Cronbach’s Alpha (α)	0.70	0.78	0.82	0.70

Dreaming that family or colleagues were infected, fear of being quarantined, worrying about increase in workload, feeling discriminated by others and being socially distanced from family and friends due to job responsibilities loaded high on factor 1; *Instability*, that reflects respondents’ fear of changes in their environment, such as imposed isolation from others and fear of heavy workload as a result of COVID-19 pandemic. Fear of being infected and of infecting others, worrying about own family becoming infected, worrying about working with COVID-19 patients and worrying about health problems in own family loaded high on factor 2; *Infection*. Factor 3; *Loss of control*, was loaded high by three items: Feeling that life is threatening, feeling of losing control of life and thinking about death/dying. Feeling insecure about whether having been infected or not, and feeling that the virus is close and may invade the body at any time loaded much on factor 4; *Concerns* (Table 4). The reliability analysis of the extracted subscales of the COVID-19 questionnaire using the Cronbach’s alpha showed high reliability (within the 0.70–0.82 region) (Table 4).

### Relationship between extracted factors and background variables and perception of preparedness

Estimates of standardized coefficients obtained from SEMs are presented in Table 5. Whether the respondents worked clinically with patients during the lockdown period or not had no bearing on any of the four latent variables (Instability, Infection, Loss of control and Concerns). However, the results showed that gender and work experience had a significant effect on Instability, Infection and Concerns. Female participants were more likely to be concerned about Instability, Infection and that the virus was close with infection being imminent. On the other hand, negative coefficients indicate that respondents with at least 10 years work experience were less likely to express fear about changes in their work-environment (Instability), fear about being infected and infecting others (Infection), and were less likely to be concerned about virus being close to them (Concerns). The same was applicable to respondents who agreed or strongly agreed that their workplace had adequate equipment and could handle both the current situation and an escalation of COVID-19 in the future well (Table 5).

**Table 5:** Standardized coefficients obtained from Structural Equation Models (SEMs) comparing professionals who worked with patients during a period when restrictions were tight with those who did not adjusted for baseline characteristics

Characteristics	Instability	Infection	Loss of control	Concerns
	β (95% CI)	β (95% CI)	β (95% CI)	β (95% CI)
Worked clinically with patients (ref: No)				
Yes	−0.01 (− 0.06, 0.09)	− 0.004 (− 0.06, 0.05)	0.03 (− 0.03, 0.09)	0.04 (− 0.01, 0.10)
Gender (ref: Male)				
Female	0.06 (0.004, 0.12) *	0.11 (0.06, 0.17)**	− 0.01 (− 0.07, 0.05)	0.07 (0.01, 0.12)*
Work experience years (ref: 0–9)				
≥ 10	− 0.13 (− 0.18, − 0.07)**	− 0.07 (− 0.12, − 0.02)**	0.002 (− 0.05, 0.06)	− 0.12 (− 0.17, − 0.07)**
Profession (ref: Dentist)				
Dental hygienist	0.01 (− 0.06, 0.09)	− 0.02 (− 0.08, 0.04)	0.04 (− 0.02, 0.10)	0.02 (− 0.04, 0.08)
Dental Assistant	0.002 (− 0.07, 0.08)	−0.01 (− 0.07, 0.05)	−0.01 (− 0.07, 0.05)	−0.04 (− 0.10, 0.02)
Size of dental clinic (ref: < 7)				
≥ 7	0.05 (− 0.02, 0.11)	0.03 (− 0.02, 0.08)	− 0.04 (− 0.10, 0.01)	0.05 (− 0.01, 0.12)
Work sector (ref: Public)				
Private	0.06 (0.003, 0.12)*	− 0.01 (− 0.07, 0.05)	− 0.03 (− 0.10, 0.03)	0.06 (− 0.001, 0.12)
County incidence of COVID-19 (ref: Low)				
Medium	0.04 (− 0.02, 0.10)	0.01 (− 0.04, 0.07)	− 0.01 (− 0.07, 0.05)	0.06 (0.003, 0.12)*
High	0.04 (− 0.01, 0.10)	0.02 (− 0.04, 0.08)	− 0.04 (− 0.11, 0.02)	0.01 (− 0.05, 0.07)
Treatment of patients suspected or confirmed to have COVID-19 (ref: Yes)				
No	0.05 (− 0.01, 0.10)	0.05 (− 0.01, 0.10)	− 0.06 (− 0.12, − 0.01)*	0.03 (− 0.02, 0.08)
Adequate equipment (ref: Other)				
Agree/ completely agree	−0.11 (− 0.17, − 0.05)**	−0.13 (− 0.19, − 0.07)**	0.15 (0.09, 0.21)**	−0.12 (− 0.18, − 0.06)**
Current situation (ref: Other)				
Agree/ completely agree	− 0.12 (− 0.18, − 0.07)**	−0.09 (− 0.14, − 0.03)**	0.09 (0.03, 0.15)**	−0.24 (− 0.54, 0.05)**
Escalation (ref: Other)				
Agree/ completely agree	−0.17 (− 0.23, − 0.11)**	−0.17 (− 0.23, − 0.11)**	0.15 (0.10, 0.21)**	−0.14 (− 0.19, − 0.08)**

*p < 0.05 and **p < 0.01 differences between dental professionals working and not working clinically with patients in the lockdown period

Our results showed that dental professionals who either agreed or strongly agreed that their workplace had adequate equipment and could handle well both current situation and any escalation of COVID-19 in the future were less likely to express feelings associated with Instability, fear about being infected or infecting others and fear that the virus could be close and infection being imminent. However, having adequate equipment in their workplace and working in an environment that could handle both current situation and any future escalation of COVID-19 did not lower Loss of control.

Despite expressing fear about the changes in their work-environment (Instability), fear about being infected or infecting others and that the virus was close and infection imminent, respondents who agreed/ strongly agreed that the risk of contracting COVID was high among dental professionals were somehow less concerned that their lives could be in danger (Loss of control).

## Discussion

The present study investigated psychological impact of the COVID-19 pandemic among dentists, dental hygienists and dental assistants in relation to perceived preparedness of dental service and work situation. The findings indicated substantial psychological burden among dental personnel in terms of fear of being infected and infecting others, regardless of working clinically with patients or not.

In early 2020, the COVID-19 pandemic resulted in a state of emergency worldwide. In the lockdown period in Norway, health personnel including dental professionals had to face sudden changes in their work situation and workload, and were forced to adapt to new and stricter routines in infection control when treating patients. At the time of lockdown, there was generally little knowledge about the SARS-CoV-2 virus. Still, urgent oral health care appeared to be managed relatively well in Norway [2]. However, most studies on psychological impact on frontline health care workers have been conducted among hospital staff, and more studies among dental staff are required.

Although COVID-19 is the first encounter with a pandemic in modern times for several countries, including Norway, other countries have experienced comparable situations in the past and have acquired knowledge that may now be beneficial for countries previously unfamiliar with large virus outbreaks. By using a questionnaire previously applied to study fear among health care workers related to severe acute respiratory syndrome (SARS), we observed that majority of dental professionals, regardless of working clinically with patients or not in the COVID-19 lockdown period, feared that they would infect others or that their family or themselves would become infected. This result is consistent with studies conducted among healthcare workers during SARS outbreak [14, 17], and among dentists during the COVID-19 outbreak [18], showing that health care workers were primarily concerned about infecting others (especially family members) and secondly concerned about being infected themselves.

A systematic review investigating the psychological impact on health care workers showed that fear of uncertainty, or fear of becoming infected, were at the forefront of the psychological challenges faced in a viral outbreak [19]. Fear of infection was high among the respondents in the current study regardless of whether the respondents worked clinically with patients or worked from home office in the lockdown period. This may suggest that in the early phase of the COVID-19 outbreak, fear of infection was generally prominent among dental professionals regardless of their work situation. Also, in the general population, the COVID-19 pandemic has been shown to be associated with highly significant levels of psychological distress [20]. In a study performed in Norway investigating the psychological and demographic factors predicting health-protective behavior in the general population during the first 2 weeks of lockdown, the participants on average reported to be moderately concerned or worried about the outbreak, while one third was very or extremely concerned [21]. Other studies have found the psychological impact of lockdowns to be small and heterogeneous, thereby suggesting that lockdowns do not affect mental health in a negative way only, and that most people are psychologically resilient to such effects [22]. However, observed high levels of fear among dental professionals are in line with most previous studies, showing fear of the unknown as a prominent stressor among healthcare workers during or following a virus outbreak [19, 23].

In line with the current study, Zhang et al. [24] compared medical and non-medical health care workers, and revealed higher levels of psychological impact among the former group. In contrast, Cawcutt et al. [25] reflected upon counterintuitive findings concerning the fear of contagion among health care workers during COVID-19 and SARS pandemics. Hospital staff not caring for infected patients reported more fear of contagion compared with frontline health care workers. The authors discussed the higher fear rates among staff at lower risk in relation to the importance of direct communication with health personnel. They argued that fear may be mitigated by direct education on the infection control measures and by addressing fear in communication with health care workers [25]. The self-perceived risk and personal fear reactions have also been shown to be associated with individual psychological differences, including personality traits [26]. However, the significance of being provided with good information and PPE to lower fear of COVID-19 remains relevant for health care workers across contexts [19].

Only a small percentage of dental professionals (less than 10%) felt that life was threatening or that they had lost control of their life. This is in contrast to a questionnaire study among doctors and nurses during the SARS outbreak, where vulnerability/loss of control and fear for self-health were among the three most important variables that could account for the distress level [27]. Higher mortality rate of SARS (10%) compared to that of SARS-CoV-2 (0.02% as of 11 Dec 2020) [1, 28] may partly explain this inconsistency.

Dental professionals’ work experience had an impact on the factors Instability, Infection and Concerns. Dental professionals with longer working experience were less likely to report fear of changes in the work-environment (heavy workload), fear of being infected and infecting others, and they were less likely to be concerned about virus being close to them. The same was applicable to respondents who perceived their workplace to have adequate equipment and handling both current situation and escalation of COVID-19 in the future well. This could be explained by increased self-confidence concerning routines among dental personnel with more work experience, as more self-confidence can generate less work-related stress. Age and clinical experience have been shown to be protective against psychological distress [29]. In contrast, the present study showed that female gender was associated with higher levels of psychological impact, as female participants were more likely to be concerned about instability, infection and that the virus was close with infection being imminent. Other studies have comparable results showing that female health workers in close contact with COVID-19 patients appeared to have the highest mental health risk [13, 26] [26]. Several explanations for such findings have been discussed, from different neurobiological responses to stress between genders [30], to different economical situations and responsibilities both at work and at home [31]. Increased distress levels in female health workers have been reported, and this could be related to an increased perception of risk both for themselves as well as for their relatives. It has also been suggested that females may be more prone to strictly adhere to rules and even to imply stricter rules than males [32].

In the present study, dental professionals who agreed or strongly agreed with the statement that dental professionals are at high risk of contracting COVID-19 (dental professionals prone to infection), were more likely to express fear of changes in their work-environment (heavy workload), fear of being infected, fear of infecting others and fear of the virus being close to them. The results suggest that working in an environment where staff feels taken care of and where infection control is adequately managed, contributes to more secure and less fearful dental professionals, even in an ongoing pandemic. This finding is supported by a study investigating the psychological impact of COVID-19 among dental health personnel in Israel, showing an association between lower psychological distress and higher self-efficacy [33]. Several studies have identified a need for greater support through collaboration, training and education during viral epidemics, to strengthen teams and reduce stress among health care workers [19]. Suggested measures that could be considered implemented by employers to minimize or prevent the psychological burden on clinical staff are clear communication, training and education around infection control procedures, enforcement of infection control procedures, adequate supplies of protective equipment and access to psychological interventions [19, 29, 34].

Confidence in services’ infection control procedures during a pandemic is also likely to decrease adverse psychological outcomes [29]. Communication and access to adequate PPE and training are suggested to be protective factors and thereby important interventions with the intend to mitigate psychological distress among front-line health care workers [19, 29]. However, during the peak of the pandemic the access to PPE was insufficient both in Norway and globally [2]. This may have contributed to an increased psychological impact among dentists in the early phase of the pandemic. It is a positive sign that Norwegian dental health personnel seemed relatively satisfied with the organization of the dental service as well as with dissemination and training. The majority of the respondents reported receiving training and simulation of step-by-step procedures both for treatment and for putting on and removing PPE. Despite this, deviations from procedures were also reported, indicating that the additional precautions needed to perform safe dental treatment in a pandemic situation are not necessarily easy to follow and require extra training and continuous follow-up.

Although most of the literature focus on psychological impact during a pandemic, detrimental effects have been reported even after an outbreak, suggesting also long-term implications [19, 35, 36]. Thus, prevention and early intervention are important to provide both contentment and stability of personnel as well as high-quality patient care. Dental health personnel are an important part of the front-line health care service and should not be neglected with regard to psychological impact of COVID-19 and concerning the significance of preventive measures. Education, training and communication with respect to COVID-19 related guidelines in infection control, followed by implementation of adequate infection control measures and a safe working environment may have a positive psychological impact on dental health personnel, and should be addressed in future studies.

### Limitations of the study

The present study was a questionnaire study, and study participants self-selected to complete the survey. Therefore, it is difficult to assess the response rate of the study and selection bias related to personal interests of clinicians may have occurred. As private practitioners were underrepresented in the study, the generalization of findings for private sector should be done with caution. The questionnaire was distributed a short while after the lockdown, and it can be argued that the timing could affect clinicians’ responses due to the rapidly changing situation. Furthermore, recall bias among participants cannot be eliminated. In general, questionnaires are prone to bias, especially regarding sensitive data, like psychological impact. However, self-administration of a questionnaire has been shown to decrease reporting bias [37]. Despite of limitations, to the best of our knowledge, the present study is of the first in Scandinavia to investigate psychological impact of COVID-19 pandemic among dental personnel.

## Conclusion

The present study showed a considerable psychological impact of the COVID-19 pandemic on dental personnel in Norway, with females, and clinicians with less working experience, reporting significantly higher impact. The results emphasize the importance of a safe working environment and implementation of proper infection control measures. This may help target specific areas that need to be addressed to reduce the psychological impact of dental professionals and prepare them better for future outbreaks. Access to adequate equipment, as well as clear communication, can be crucial in reducing fear of infection and the feeling of instability in the dental professionals when working with patients in a pandemic outbreak. It is reasonable to assume that increased knowledge of COVID-19 features, epidemiologic characteristics, and preventive strategies may result in reduced negative psychological impact of the pandemic among dental personnel.

## Data Availability

The dataset used in the current study is available from the corresponding author on reasonable request.
